# Descriptive study of claims for occupational mental disorders or suicide

**DOI:** 10.1186/s40557-016-0147-7

**Published:** 2016-10-20

**Authors:** Jihoon Lee, Inah Kim, Sooyong Roh

**Affiliations:** 1 Department of Occupational and Environmental Medicine, Hanyang University Medical Center, 222-1, Wangsimni-ro, Seongdong-gu, Seoul 04763 Korea; 2 Department of Occupational and Environmental Health, College of Medicine, Hanyang University, 222 Wangsimni-ro, Seongdong-gu, Seoul 04763 Korea

**Keywords:** Mental disorder, Suicide, Claimed data

## Abstract

**Background:**

This study aimed to identify the characteristics of claimed mental disorders. Because the workers believed the cause of the mental disorders was work-related stress or a specific event, we could identify the major work-related stressor for claimed cases.

**Methods:**

We included claimed cases of occupational mental disorder or suicide reported during 2010–2014 to the Korea Workers Compensation and Welfare Service (KCOMWEL), established by Industrial Accidents Insurance (IACI) Act. We conducted qualitative analysis using a form specifically developed for this study as well as a quantitative analysis.

**Results:**

Of the 569 claimed cases, 142 cases were recognized as occupational mental disorder or suicide. The approval rate was 24.9 %. Suicide was the most commonly approved mental disorder (23.0 %), followed by major depressive disorder (14.9 %). Regarding profession, 109 workers were managers, and 95 workers were office clerks. The main work-related stressors of the approved cases were acute stressful events (76 cases), long working hours (12 cases), and changes in workload (6 cases). The primary stressful events were work-related legal problems, workplace violence, and employment status-related issues.

**Conclusion:**

Claims due to mental disorders or suicide increased during the 5-year study period, and the approval rate was approximately 33 %, and the main stressor of the claimed cases was an acute stressful event such as physiologic trauma, employment-related issues, fear of legal or financial responsibility, abrupt change in organizational responsibility, or workplace violence.

## Background

Mental disorders are a main cause of loss of productivity and global burden of disease [1, 2]. Recently, researchers and policy makers have become interested in suicides and mental disorders related with working conditions such as long working hours or low wages; psychosocial factors such as job strain, unemployment, job instability, or emotional demand; and workplace violence from a third party or supervisor in South Korea [3–6].

In 2013, the Ministry of Employment and Labor (MOEL) in Korea amended the Enforcement Decree of Industrial Accident Compensation Insurance (ED-IACI) Act and added post-traumatic stress disorder (PTSD) due to work-related psychological trauma [7]. In 2016, the MOEL amended the ED-IACI Act again and added depressive episodes or adjustment disorders due to third-party workplace violence or directly related stress [8].

In South Korean, the recognition process for all of the mental disorder including PTSD was same with the process of occupational disease. At first, the Korea Workers’ Compensation and Welfare Service (COMWEL) conducts investigations about the exact diagnosis, working condition, work stress, symptoms and signs, medical records, or individual risks. Finally, Committee on Occupational Disease Judgement (CODJ) composed with occupational physician, psychiatrist, or law expertise decided whether the disease compensated with IACI or not [9].

According to the Epidemiologic Survey of Mental Disorders of the Ministry of Health and Welfare, in 2011, the lifetime prevalence of major mental disorders was 27.6 %, of mood disorder were 3.6 %, and of anxiety disorders was 6.0 % in adults aged 18–74 years [10]. However, the specific situations related with or prevalence of workers’ mental disorders is unknown.

This study aimed to identify the characteristics of and major work-related stressors related with claimed mental disorders, which the workers believed to be caused by work-related stress or a specific event.

## Methods

### Subjects

We included all of the claims cases for 2010–2014 from the Korea Workers Compensation and Welfare Service (KCOMWEL). We only included the cases which was not the complicated disease, claimed from 2010 to 2014, and the final decision for the approval or not was ended in April 2015.

### Qualitative analysis

The KCOMWEL provided all of the data for the claims, including various forms of evidence for the main occupational stressor or event that was claimed against; working history; job characteristics; working condition; various job stressors; interviews with the workers, subscribers, coworkers, or employers; diagnosis; medical records; suicide notes; diaries; cell phone messages; official investigation result; and decision statement from the CODJ. We developed a typical classification form to identify the main recent stressor, underlying stress, individual characteristics, past history, and involvement in emotional labor from the documents. We defined ‘emotional labor’ as the main task of workers should directly contact with customer or human and we decided that according to the detailed job classification and the explanation of their task process. We also review the CODJ decision report to identify the main reasons of approval of disapproval. We constructed a data set for the identified cases for the 5-year period.

### Quantitative analysis

We analyzed the frequencies of the descriptive characteristics.

## Results

We identified 596 cases of mental disorders or suicide (Table 1). Men accounted for 74.8 % (*n* = 426) of the cases, and women accounted for 25.2 % (*n* = 143) of the cases. Regarding age, 38.8 % (*n* = 221) were aged 40–49 years, 24.4 % (*n* = 139) were aged 30–39 years, and 22.8 % (*n* = 130) were aged 50–59 years. Regarding roles, 20.3 % were involved in emotional labor, 20.6 % of the men were managers, and 27.5 % of the women were office clerks.

**Table 1:** General characteristics of the cases of mental disorders or suicide in the claims database

	Total	Men	Women
Age (years)			
< 30	54 (9.5)	41 (9.6)	13 (9.2)
30–39	139 (24.4)	77 (22.7)	42 (29.6)
40–49	221 (38.8)	180 (42.2)	41 (28.9)
50–59	130 (22.8)	92 (21.5)	38 (26.8)
≥ 60	25 (4.4)	17 (4.0)	8 (5.6)
Alcohol consumption			
Current drinker	249 (43.8)	211 (49.4)	38 (26.8)
Ex-drinker, Never-drinker	320 (56.2)	216 (56.2)	104 (73.2)
Smoking			
Current smoker	199 (35.0)	189 (44.3)	10 (7.0)
Ex-smoker, Never-smoker	370 (65.0)	238 (55.7)	132 (93.0)
Emotional labor			
Yes	110 (20.3)	72 (16.9)	38 (26.8)
No	433 (79.7)	355 (83.1)	78 (54.9)
Occupation			
Craft and related trade workers	12 (2.1)	10 (2.3)	2 (1.4)
Engineer or technician	56 (9.9)	48 (11.2)	8 (5.6)
Elementary worker	70 (12.3)	61 (14.3)	9 (6.3)
Clerk	95 (16.8)	56 (13.1)	39 (27.5)
Service worker	71 (12.5)	44 (10.3)	27 (19.0)
Manager	109 (19.2)	88 (20.6)	21 (14.8)
Equipment operator, machine operator, or assembly line worker	93 (16.4)	82 (19.2)	11 (7.7)
Professional and related worker	37 (9.5)	25 (5.9)	12 (8.5)
Other occupation	5 (0.9)	3 (0.7)	2 (1.4)
Sales worker	19 (3.4)	10 (2.3)	9 (6.3)
Total	569 (100.0)	427 (75.1)	142 (24.9)

Values are reported as *n* (%)

The approval rate over the 5-year period was 33.2 %, and the number of claims increased from 83 cases in 2010 to 135 cases in 2014 (Table 2).

**Table 2:** Number of workers' compensation certifications and approval rates for mental illness, by year

	Final approval		Total
	Approval	Disapproval	
2010	21 (25.3)	62 (74.7)	83 (14.6)
2011	29 (29.3)	70 (70.7)	99 (17.4)
2012	35 (36.9)	77 (63.1)	122 (21.4)
2013	48 (36.9)	82 (63.1)	130 (22.8)
2014	46 (34.1)	89 (65.9)	135 (23.7)
Total	189 (33.2)	380 (66.8)	569 (100.0)

Values are reported as *n* (%)

The most common claim was suicide (23.0 %, *n* = 203), followed by depression (14.9 %, *n* = 132), adjustment disorder (9.6 %, *n* = 85), and PTSD (7.2 %, *n* = 64). Other disorders included systemic disorders such as musculoskeletal disorders, heart disorders, or gastrointestinal disorders, which are not mental disorders.

The approval rates for adjustment disorder, acute stress disorder, and PTSD were relatively high (Table 3).

**Table 3:** Disease-specific approval rates for mental illness

Diagnosis (Duplicates were allowed)	Final approval		Total
	Approval	Disapproval	
Suicide	74 (36.5)	129 (63.5)	203 (23.0)
Depression	48 (36.3)	84 (63.6)	132 (14.9)
Adjustment disorders	41 (48.2)	44 (51.8)	85 (9.6)
PTSD	41 (64.1)	23 (35.9)	64 (7.2)
ASD	34 (68.0)	16 (32.0)	50 (5.7)
Sleep disorders	24 (53.3)	21 (46.7)	45 (5.1)
Psychosis	2 (4.9)	39 (95.1)	41 (4.6)
Anxiety disorders	20 (45.0)	38 (55.0)	58 (4.5)
Panic disorder	7 (17.9)	32 (82.1)	39 (4.4)
Mood disorders^a^	2 (11.1)	16 (88.9)	18 (2.0)
Other mental disorders	3 (11.5)	23 (88.5)	26 (2.9)
Total cases	294 (39.6)	449 (60.4)	743 (100.0)

Values are reported as *n* (%)

*PTSD* post-traumatic stress disorder, *ASD* acute stress disorder

^a^mood disorder were not classified the exact diagnosis according to ICD (Internatinal classification of Disease) -10 code due to lack of Diagnosis Certification

Table 4 shows the relationship between the approval rate and stress due to emotional labor or workplace violence. The approval rate for workers with a main job task that involved emotional labor (31.8 %) was not different from that for workers not involved in emotional labor (33.3 %). The approval rate for a history of exposure to workplace violence for other workers whose main job task did not involve emotional labor was 44.7 %.

**Table 4:** Claim approval rate based on emotional labor or workplace violence

	Final approval		
	Approval	Disapproval	Total
Emotional labor	35 (31.8)	75 (68.2)	110 (20.3)
Nonemotional labor	144 (33.3)	289 (66.7)	433 (79.7)
Total	179 (34.0)	364 (67.0)	543 (100.0)
Exposed to violence	51 (44.7)	96 (65.3)	147 (32.0)
Never	99 (31.7)	213 (68.3)	312 (68.0)
Total	150 (32.7)	309 (67.3)	459 (100.0)

Values are reported as *n* (%)

The main reasons for approval or disapproval are shown in Table 5. An acute stressful event was the most common reason for approval (56.3 %). Chronic long working hours (8.9 %), change in materials or personal resources (5.2 %), and change in workload (4.4 %) were also important stressors reported in the claims. The most common reason for disapproval was low stress intensity (38.1 %), and 16.9 % of claims were not approved because the main stress was related with a personal condition. An ambiguous diagnosis was also an important reason for disapproval.

**Table 5:** The main reasons of approval or disapproval decision

Main reasons (Duplicates were allowed)	Number	Percent
Approval	135	(100.0)
Acute stressful event	76	(56.3)
Chronic long working hours	12	(8.9)
Change in workload	6	(4.4)
Change in work quality	2	(1.5)
Change in responsibility	5	(3.7)
Lack of discretionary authority	2	(1.5)
Change in materials or personal resources	7	(5.2)
Organizational support	2	(1.5)
Emotional labor	1	(0.7)
Others	3	(2.2)
Disapproval	353	(100.0)
Low stress intensity	122	(38.1)
Personal stress	54	(16.9)
Aggravation of personal mental disorder	66	(20.6)
Others^a^	78	(24.4)
Total	488	(100.0)

^a^Diagnosis was not confirmed, clinically presumed diagnosis, duplicated diagnosis, unmet diagnosis criteria, lack of direct causal association, no objective sign, loss of stress evidence, illegal violence due to claim obligation relationship etc

## Discussion

There was an increase in claims due to mental disorders or suicide during the 5-year period. The approval rate was approximately 33 %, and the main stressor was an acute stressful event, including physiologic trauma, employment-related issues, fear of legal or financial responsibility, abrupt change in organizational responsibility, or workplace violence.

Regarding suicide, fear of legal or financial responsibility was a reported work-related stressor; this is also related with economic problems. The second stressor was responsibility related with an ethical problem or qualification; examples include an inspection by the Prime Minister’s Office, sudden progression in actual or acting authority, sudden change in supervisor or task, pressure to solve a socially-focused issue (e.g., environmental disaster), unexpected transfer, or competitive performance evaluation. Another main stressor was interpersonal relationships related with mobbing, bullying, or workplace violence. Finally, employment-related issues such as a transfer without agreement, resignation, notice of discharge, or termination of contract were important causes of suicide or mental disorders.

Managerial and office work were the primary main occupation of the workers with a claim. This is very different from the occupations reported for other occupational disorders or occupational injuries. Blue collar were usually main occupations for occupational injuries or diseases [11]. This could be related with financial or legal responsibility, which is also related with economic problems and labor market characteristic of labor market; for example, 62.3 % of employees are white collar or service workers [12].

Adjustment disorder, acute stress disorder, and PTSD were commonly approved disorders, while approvals were typically not granted for panic disorder or schizophrenia. The first three disorders are caused by external stress or trauma, as classified in the Diagnostic and Statistical Manual of Mental Disorders, Fifth Edition (DSM-V) [13]. In contrast, panic disorder or schizophrenia is considered to be caused by intrinsic factors.

IACI does not compensate for suicide or self-harm, in principle. When the injury, disease, disability, or death is caused by any act committed in the state of a marked decline in his/her normal cognitive function, as prescribed by Presidential Decree, it is deemed an occupational disease [14]. The three exceptional cases include self-harm in a state of mental disorder by a person who received or is receiving medical treatment for a mental illness arising from work-related reasons; by a person who is receiving medical care due to a work-related accident, and the mental disorder is caused by the work-related accident; and by a person due to work-related reasons, when the relationship between the reason and self-harm is medically recognized [14]. Therefore, regarding the approval rate of approximately 36 %, we did not attempt to determine a causal association between stress and suicide or compare the figure with that from other countries. However, considering that there was an increase in claimed cases and the suicide rate among employees was very high in Korea, various epidemiologic study to identify cause, trigger, underline risk factor, or high risk group of suicide, for example case–control study using psychological autopsy, cohort study, or other study using various research methods are also needed.

KCOMWEL provided all documents related to claimed cases. Therefore, the documents may have mainly included the employee’s or their family’s subjective opinion or theirs ideas depending on how much they remember. This suggests that all study subjects want to approve of their claim and the findings of this study may not be representative of the status, occupational stressors, or mental disorders or suicides among all workers. We also analyzed official documents and statements from KCOMWEL and CODJ.

We conducted text review by developing a common form for descriptive analyses but we could not verify the validity and reliability of this form. However, only three occupational physicians conducted the text review and one physician reviewed all data after the first review.

## Conclusions

To our knowledge, this is the first descriptive study to investigate the trends and issues related with occupationally claimed mental disorders and suicides among employees. We used qualitative and quantitative methods to identify the main claimed cause of mental disorders and suicides. The findings are few but are important as fundamental data that are be used by future studies.

## Abbreviations

CODJ: Committee of Occupational Disease Judgment
DSM-V: Diagnostic and Statistical Manual of Mental Disorders, Fifth Edition
ED-IACI: Enforcement Decree of Industrial Accident Compensation Insurance
KCOMWEL: Korea Workers Compensation and Welfare Service
MOEL: Ministry of Employment and Labor
PTSD: Post-traumatic stress disorder

## References

[1] Schofield DJ, Shrestha RN, Percival R, Passey ME, Callander EJ, Kelly SJ (2011). The personal and national costs of mental health conditions: impacts on income, taxes, government support payments due to lost labour force participation. BMC Psychiatry.

[2] Whiteford HA, Degenhardt L, Rehm J, Baxter AJ, Ferrari AJ, Erskine HE, Charlson FJ, Norman RE, Flaxman AD, Johns N, Burstein R, Murray CJ, Vos T (2013). Global burden of disease attributable to mental and substance use disorders: findings from the Global Burden of Disease Study 2010. Lancet.

[3] Min JY, Park SG, Kim SS, Min KB (2014). Workplace injustice and self-reported disease and absenteeism in South Korea. Am J Ind Med.

[4] Lee G (2015). Korean emotional laborers' job stressors and relievers: focus on work conditions and emotional labor properties. Saf Health Work.

[5] Jang SY, Jang SI, Bae HC, Shin J, Park EC (2015). Precarious employment and new-onset severe depressive symptoms: a population-based prospective study in South Korea. Scand J Work Environ Health.

[6] Lee KJ, Kim JI (2015). Relating factors for depression in Korean working women: secondary analysis of the fifth Korean National Health and Nutrition Examination Survey (KNHANES V). Asian Nurs Res (Korean Soc Nurs Sci).

[7] Kang DM, Kim I (2014). Compensation for occupational neurological and mental disorders. J Korean Med Sci.

[8] Sung-jin C (2016). Workers with depression can claim compensation. The Korea Times.

[9] Kwon SC, Kim HR, Kwon YJ (2014). The administrative process for recognition and compensation for occupational diseases in Korea. J Korean Med Sci.

[10] Cho MJ, Seong SJ, Park JE, Chung IW, Lee YM, Bae A, Ahn JH, Lee DW, Bae JN, Cho SJ, Park JI, Son J, Chang SM, Hahm BJ, Lee JY, Sohn JH, Kim JS, Hong JP (2015). Prevalence and correlates of DSM-IV mental disorders in South Korean adults: the Korean epidemiologic catchment area study 2011. Psychiatry Investig.

[11] Won J, Ahn Y, Song J, Koh D, Roh J (2007). Occupational injuries in Korea: a comparison of blue-collar and white-collar workers' rates and underreporting. J Occup Health.

[12] Jeon SH, Leem JH, Park SG, Heo YS, Lee BJ, Moon SH, Jung DY, Kim HC (2014). Association among working hours, occupational dtress, and presenteeism among wage workers: results from the second Korean Working Conditions Survey. Ann Occup Environ Med.

[13] DSM-5 Table of contents. In: DSM-5 Resources. https://www.psychiatry.org/psychiatrists/practice/dsm/dsm-5. Accessed 25 July 2016.

[14] Reliable Industry of Government Legislation: National Law Information Center. http://www.law.go.kr/eng/engLsSc.do?menuId=1&query=%EC%82%B0%EC%97%85%EC%9E%AC%ED%95%B4%EB%B3%B4%EC%83%81%EB%B3%B4%ED%97%98%EB%B2%95&x=0&y=0#liBgcolor16 (2015). Accessed 25 July 2016.

